# Long COVID and Associated Factors Among Chinese Residents Aged 16 Years and Older in Canada: A Cross-Sectional Online Study

**DOI:** 10.3390/biomedicines13040953

**Published:** 2025-04-13

**Authors:** Matin Shariati, Kieran Luke Gill, Mark Peddle, Ying Cao, Fangli Xie, Xiao Han, Nan Lei, Rachel Prowse, Desai Shan, Lisa Fang, Vita Huang, Arianna Ding, Peizhong (Peter) Wang

**Affiliations:** 1 Division of Population Health and Applied Health Sciences, Faculty of Medicine, Memorial University, 300 Prince Philip Drive, St. John’s, NL A1B 3V6, Canada; dr.matin.shariati@gmail.com (M.S.); klgill@mun.ca (K.L.G.); mep407@mun.ca (M.P.); leinan139@gmail.com (N.L.); rprowse@mun.ca (R.P.); dshan@mun.ca (D.S.); lisafang.f@gmail.com (L.F.); 2 Centre for New Immigrant Well-Being (CNIW), 96 Scarsdale Road, Toronto, ON M3B 2R7, Canada; info@cniw.org (Y.C.); sarahxie2012@gmail.com (F.X.); hanxiao528@gmail.com (X.H.); huangvita36@gmail.com (V.H.); dingarianna46@gmail.com (A.D.); 3 Dalla Lana School of Public Health, University of Toronto, 155 College Street, Room 534, Toronto, ON M5T 3M7, Canada

**Keywords:** COVID-19, long COVID, immigrant health, long COVID risk factors, Chinese immigrants, Canada, LASSO regression

## Abstract

As the COVID-19 pandemic evolved, long COVID emerged as a significant threat to public health, characterized by one or more persistent symptoms impacting organ systems beyond 12 weeks of infection. Informative research has been derived from assessments of long COVID among the Chinese populace. However, none of these studies considered the COVID-19 experience of Chinese residents in Canada. **Objectives:** We aimed to fill this literature gap by delineating the long COVID experience, prevalence, and associated factors among a sample of Chinese residing in Canada during the pandemic. **Methods**: The present study employed a cross-sectional online survey questionnaire distributed to a sample of Canadian Chinese using a convenience sampling procedure from 22 December 2022 to 15 February 2023. Respondents were probed for sociodemographic background and health-, COVID-, and vaccine-related characteristics. Logistic LASSO regression was used for model building, and multivariate logistic regression was used to identify factors associated with developing long COVID. **Results**: Among 491 eligible participants, 63 (12.83%) reported experiencing long COVID with a mean duration of 5.31 (95% CI: 4.06–6.57) months and major symptoms including difficulty concentrating (21.67%), pain/discomfort (15.00%), as well as anxiety/depression (8.33%). Our final model identified significant associations between long COVID and two or more COVID-19 infections (OR = 23.725, 95% CI: 5.098–110.398, *p* < 0.0001), very severe/severe symptoms (OR = 3.177, 95% CI: 1.160–8.702, *p* = 0.0246), over-the-counter medicine (OR = 2.473, 95% CI: 1.035–5.909, *p* = 0.0416), and traditional Chinese medicine (OR = 8.259, 95% CI: 3.016–22.620, *p* < 0.0001). Further, we identified a significant protective effect of very good/good health status (OR = 0.247, 95% CI: 0.112–0.544, *p* = 0.0005). **Conclusions**: Long COVID effected a notable proportion of Canadian Chinese for a prolonged period during the COVID-19 pandemic. Our findings underscore the importance of preexisting health status and reinfection prevention when managing long COVID. Moreover, our work indicates an association between using over-the-counter medicine or traditional Chinese medicine and long COVID experience among Canadian Chinese.

## 1. Introduction

The World Health Organization (WHO) declared coronavirus disease 2019 (COVID-19) a Public Health Emergency of International Concern (PHEIC) on 30 January 2020 and a pandemic on 11 March 2020 [1]. According to WHO, as of 15 March 2025, over 777 million people have been infected, and more than 7 million people have died globally [2]. During this period Canada reported around 4.8 million confirmed COVID-19 cases and more than 55,000 deaths. Within Canada, the Greater Toronto Area’s (GTA) first coronavirus cases came from mainland China in January 2020, as did most COVID-19 infections during the early pandemic [3]. According to 2021 census records, the population of Canadian Chinese residents was around 1.7 million, or approximately 4.7% of the total population [4]. This makes Canadian Chinese one of the largest immigrant populations in Canada. Notably, compared to other Canadians, Chinese immigrants have more frequent and intimate relationships with China, where the first cases of COVID-19 were detected, making them uniquely susceptible to infection as COVID-19 spread around the world [5].

COVID-19 targets respiratory systems, but research indicates other organs are also affected. Symptoms associated with lower respiratory tract infection, including fever, dry cough, and difficulty breathing, were reported in the first case series in Wuhan, China [6]. Further, headache, dizziness, weakness, vomiting, and diarrhea have also been reported [7]. Major complications reported in patients with COVID-19 have also included coagulopathy, laryngeal edema, laryngitis, necrotizing pneumonia, acute respiratory failure, ventilation-associated pneumonia, massive pulmonary embolism, sepsis, and a higher risk of mortality [8]. Additionally, long COVID may occur after experiencing a wide variety of mild to severe COVID-19 symptoms [9]. Long COVID is a symptom that continues or develops beyond 12 weeks from an acute COVID-19 infection, with no explanation from any other diagnosis [10]. Studies have shown respiratory, cardiovascular, neurological, gastrointestinal, and musculoskeletal systems are affected by long COVID [9,11,12]. The most common symptoms include fatigue, cough, sore throat, dyspnea, cardiac abnormalities, sleep turbulences, myalgia, arthralgia, cognitive impairment, concentration problems, and headaches [9]. Interestingly, cognitive impairment and mental health issues, including “brain fog”, anxiety, post-traumatic stress disorder, depression, and sleep disturbances are common and debilitating long-term effects of COVID-19 infection. Research suggests neuroinflammation, vascular damage, and psychological stressors contribute to neuropsychiatric symptoms, particularly among individuals with severe symptoms, hospitalization, and prolonged ventilation.

Importantly, COVID-19 infection has been shown to induce production of antibodies able to damage proteins, leading to autoimmune cell damage that could persist following initial illness [13]. Further, research has identified additional potential pathophysiological mechanisms underlying long COVID. These investigations highlight the occurrence of hypercoagulation, characterized by the formation of micro-clots and endothelial dysfunction. Concurrently, disruption of cellular energy metabolism has been identified as a contributing factor, ultimately culminating in a hypoxic state [14].

Considering the significant impact of long COVID on individual health and at-risk groups, it is crucial to develop and implement targeted preventative measures to mitigate the risk of developing long COVID, in addition to efforts to prevent initial infection. Numerous risk factors for developing long COVID have been identified in research conducted on the Chinese populace. More specifically, the risk factors most closely linked to long COVID include being female [15,16,17,18], experiencing many initial symptoms of COVID-19, having increased levels of D-dimer and C-reactive protein (CRP), having prior psychiatric disorders, engaging in military and transport jobs, smoking, reporting poor self-perceived health status, having chronic diseases or using medication, having early dyspnea, and experiencing critical severity of COVID-19 [19,20].

In combating the health impacts of COVID-19, various vaccines were created as the pandemic evolved. A comparison of long COVID symptoms between unvaccinated and vaccinated individuals has indicated a robust correlation between vaccination and reducing long COVID symptoms [21,22,23]. That is, vaccination reduced acute COVID-19 infection severity, which reduced long COVID symptoms. However, the consequence of vaccination in people with long COVID symptoms remains unknown and controversial [24]. For example, one study showed the severity of COVID-19 symptoms improved more in those vaccinated with mRNA-type vaccines than those immunized with an adenovirus vector [25]. Similarly, a systematic review study presented that the effect of vaccination on long COVID symptoms was supported in some studies and not in other studies, which could be due to the variation in the type of vaccine received in different studies [24].

Traditional Chinese beliefs about health and illness are distinct from those of Western societies. For instance, one study exploring the health beliefs of older Chinese Australians found they possessed a holistic view of health and the role of food in preventive care and self-medication in times of illness [26]. Another study found some Chinese residents of the United Kingdom prefer the self-treatment approach of traditional Chinese medicine (TCM) or use TCM when they feel Western medicine offers ineffective treatment [27]. Overall, social images of health and illness are closely linked to cultural identity [28]. Chinese have less desire to integrate into Canadian society, which may make them less willing to seek health services.

To date, although long COVID has been explored in numerous countries, considering diverse factors, most studies assessing the COVID-19 experiences of Chinese individuals have been in the Chinese context [20,29,30]. That is, there is a notable gap in research pertaining to the COVID-19 experience of Chinese residents in Canada. Most importantly, no large-scale study has been conducted on Canadian Chinese regarding long COVID [5]. Accordingly, the current study aims to address the following research question: what are the demographic, medical, and behavioral factors (i.e., sociodemographic, COVID-, health-, and vaccine-related factors) associated with the likelihood of developing long COVID among Chinese residents in Canada during the global COVID-19 pandemic? More specifically, this study addresses two specific objectives: (1) to define and contrast the characteristics of a subset of Chinese residents in Canada in relation to their long COVID experience; (2) to evaluate any associations between the characteristics of Canadian Chinese and development of long COVID. Identifying the factors associated with long COVID among Canadian Chinese could help health professionals and policymakers better understand the unique needs and vulnerabilities of East Asian Canadians, and particularly Chinese residents in Canada. Characterizing the long COVID experience of these vulnerable Canadians is important for planning rehabilitative services that support an efficient return to typical daily activities (e.g., social, academic, vocational) [31,32]. Furthermore, such information may contribute foundational knowledge prerequisite for adequate policies, programs, and services supporting rapidly growing Canadian immigrant populations during future communicable disease outbreaks [33].

## 2. Materials and Methods

### 2.1. Study Design and Setting

Our cross-sectional online survey questionnaire was distributed among a sample of Canadian Chinese from 22 December 2022 to 15 February 2023. During this period a multifaceted public health response to COVID-19 was still ongoing, prominently including restricted contact between people (e.g., lockdowns), travel restrictions, and widespread vaccination against COVID-19 [34]. All methodologies employed in the current study strictly adhere to all pertinent guidelines and regulations.

To estimate effective sample size, we used the Cochrane formula, considering the total Chinese population in Canada (1.7 million), an assumed long COVID prevalence of 10%, confidence coefficients of 95%, precision level of 5%, and 10% dropout [35,36]. We estimated 425 for the effective sample size. Only individuals of self-identified Chinese ethnicity residing in Canada were invited to complete the survey questionnaire. This included Canadian citizens and permanent residents of Chinese ancestry, international students, and individuals holding valid work permits [37,38]. Additional eligibility criteria included individuals 16 years of age or older and living in Canada for at least 6 months at the time of survey questionnaire completion. Our online survey was voluntarily completed by 591 respondents, with nearly 85% of respondents (N = 502) meeting all eligibility criteria for inclusion in this study. Among these valid responses, 491 respondents with completed age group and immigration status measures were included in final data analyses (see Supplemental Material—S1).

Utilizing a convenience sampling procedure, our survey questionnaire was distributed by leveraging communication channels widely used by Canadian Chinese, including social media platforms such as WeChat, emails, and community organizations such as the Consulate General of China in Toronto, Ontario. Potential respondents were provided with a brief study overview and a consent form detailing principles of anonymity and their rights as participants. Subsequently, informed consent was obtained from all participants involved in the study prior to beginning the survey questionnaire. Further, respondents had the option to withdraw their participation at any time before clicking “Submit” to record their survey questionnaire responses. After submitting survey responses, participants could enter their email in an incentive draw with a CAD 25.00 electronic shopping card (10 in total). Utilizing IP addresses, we deterred duplicate entries and fostered genuine responses. To safeguard privacy, all identifiers, such as WeChat IDs, email addresses, and IP addresses, were removed and not associated with responses.

For all data collection, we utilized an online survey questionnaire created in Qualtrics™ and made available in traditional Chinese, simplified Chinese, and English to ensure accessibility to a sufficiently large sample of Canadian Chinese. This questionnaire was also used in previous studies conducted on Canadian Chinese [39,40,41]. Following informed consent, the questionnaire commenced with questions screening for each eligibility criterion and then proceeded through five sections.

The first section aimed to gather background and general sociodemographic information about Chinese in Canada. Next, the second section focused on experiences of individuals with COVID-19 infection, symptom severity after the most recent infection, COVID-19 infection prevention efforts, and types of treatment. In the third section, participants were asked about experiences with COVID-related symptoms more than 12 weeks after being infected with COVID-19. The fourth section contained questions about current health status, diagnosis with underlying diseases, risky health behaviors (e.g., smoking status, regular alcohol consumption), and other health behaviors (e.g., utilizing supplements, traditional Chinese medicine). Additionally, the fifth section explored information on the participants’ COVID-19 vaccination history, COVID-19 vaccine type, side effects after receiving the COVID-19 vaccine, and past year Influenza vaccination status.

### 2.2. Main Outcome Variable—Long COVID

One question assessed experienced long COVID: “Some people still have physical or psychological symptoms more than 12 weeks after being infected with the coronavirus. This phenomenon is called long COVID. Do you think you have or have had long COVID symptoms?” A binary outcome variable was created, with responses “Yes, recovered now” and “Still experiencing long COVID-19” classified as “Yes” and “No” classified as “No”.

### 2.3. Long COVID Experience

The duration of any long COVID symptoms experienced by participants was reported as a continuous interval variable with a unit of months. Symptoms of long COVID were reported as “Fatigue”, “Memory problems”, “Sleep disorder”, “Shortness of breath”, “Anxiety and depression”, “Pain/discomfort”, “Difficulty thinking or concentrating”, and “Others”. The question assessing symptoms of long COVID allowed for multiple selections. Additionally, participants who selected the response option “Others” (n = 24) were prompted to specify additional symptoms via a text box entry. These variables related to long COVID experiences were not included in logistic regression models, as they applied only to participants reporting a history of long COVID.

### 2.4. Covariates

We measured 20 covariates across five broad categories. For improved data analysis and more efficient interpretation, response categories with no or limited data were reclassified into closely related or otherwise appropriate categories. Similarly, questions with too many categories were also reclassified by merging related or similar categories.

#### 2.4.1. Background and General Sociodemographic Information

Age groups responses included “Under 25”, “25 to 34”, “35 to 44”, “45 to 54”, “55 to 64”, and “65 or older” and were reclassified as “Under 45”, “45 to 64”, and “65 or older. For logistic regression analyses, age group was dummy coded, using “Under 45” as the reference category. Gender comprised “Female”, “Male”, and “Others”, reclassified as “Men” and the majority, “Women”, including “Others” (n = 3). Religiosity responses were reclassified as “Not religious” for “None” and “Religious” for “Christianity”, “Catholicism”, “Islam”, “Buddhism”, and “Others”. Marital status comprised “Single/divorced/widowed” and the majority, “Married/common law”, merged with “Others” (n = 2).

Employment as a health care worker and public contact at work were both assessed as “Yes” and “No”. Financial satisfaction was assessed on a 5-point Likert scale and reclassified as “Satisfied” for “Satisfied” or “Very satisfied” and “Not satisfied” for “Neutral”, “Dissatisfied”, or “Very dissatisfied”. Province was assessed as “Ontario”, “Quebec”, “British Columbia”, “Alberta”, “Saskatchewan”, “Manitoba”, “Atlantic Canada”, and “Other provinces of Canada”, then reclassified as “Ontario” and “Others”.

Presence of children under the age of 16 in the household was evaluated as “No”, “Yes–one”, “Yes–two”, and “Yes–three or more” (n = 4), merged into “No”, “Yes–one”, and “Yes–two or more”. Presence of adults aged 65 or above in the household comprised “No”, “Yes–one”, “Yes–two”, “Yes–three or more” (n = 1), merged into “No”, “Yes–one”, and “Yes–two or more”. For logistic regression analyses, these two measures were combined, creating a new household composition variable, categorized as “Yes” and “No”.

#### 2.4.2. COVID-19 Infection Experiences

Number of positive COVID-19 test results were measured as “None”, “Infected once”, “Infected twice”, “Infected more than twice”, and “Not sure”, later reclassified as “None/not sure”, “One”, and “Two or more”. For participants who were not sure or reported at least one positive COVID-19 test result, COVID-19 symptom severity during their most recent COVID-19 infection was assessed on a 5-point Likert scale, later merged into “Asymptomatic/very mild”, “Mild”, and “Serious/very serious”. For logistic regression, COVID-19 symptom severity was dummy coded with “Asymptomatic/very mild” as the reference. Next, treatment received for most recent COVID-19 infection was assessed as “Did not treat symptoms”, “Traditional Chinese medicine”, “Over-the-counter medicine”, and “Prescription medicine”, with “Prescription medicine” later merged with “Hospitalized” (n = 3). Once again, for logistic regression analyses, COVID-19 treatment was dummy coded, using “No treatment” as the reference category.

#### 2.4.3. Health and Health Behaviors

Health status was assessed using a 5-point Likert scale reclassified as “Very good/good” and “Fair/poor/very poor”. Underlying health conditions comprised “Hypertension”, “Diabetes”, “Heart disease”, “Allergy”, “COPD”, “Asthma”, “Arthritis”, “Osteoporosis”, “Cancer”, “Back pain/lumbar pain”, “Obesity”, “Others”, and “None”. This measure allowed for multiple selections. A binary underlying health conditions variable was then created, with categories including “One or more” and “None”. Smoking status was reclassified as “Smoker” for “Yes” and “Occasionally” or “Non-smoker” for “Not willing to answer” (n = 2) merged with the majority, “No”. Similarly, regular alcohol consumption was reclassified as “Yes” for “Yes” and “Occasionally” or “No” for “Not willing to answer” (n = 2) merged with the majority, “No”.

#### 2.4.4. Vaccination History

COVID-19 vaccination history was measured as “Never”, “Once”, “Twice”, and “Three or more” for “Three times” and “Four or more times”. Side effects after receiving COVID-19 vaccination were recategorized as “Yes” and “No/not sure”. Finally, past year Influenza vaccination status was reclassified as “Yes” and the majority, “No”, merged with “Not sure” (n = 2).

### 2.5. Missing Data

Prior to analyzing the final dataset, we explored missing data frequency and proportion by observation across the final dataset. The pattern and extent of missingness reflected an arbitrary pattern of missingness. Observed data was leveraged to estimate the likelihood of missingness for each covariate and its association with other variables considered in explanatory analyses [42]. Upon inspecting the entire dataset and exploring associations between covariates and missing values in each variable, we assumed data in the study were missing at random. Accordingly, we handled all missing data through multiple imputations by fully conditional specification, a valid approach used to handle missing data across categorical variables [43,44].

For the final sample, there was a missing data rate of 0.61% to 21.38% for variables considered in explanatory analyses, with an overall missing data rate of 8.48%. This indicates the potential for multiple imputation to reduce bias and improve validity of parameter estimates for the associations of interest [42,44]. In accordance with the overall proportion of missing values in our dataset, we generated 10 imputed datasets for analysis [44]. Notably, there were no missing values for the main outcome variable, long COVID, or covariates including age group, marital status, and COVID-19 symptom severity.

### 2.6. Statistical Analyses

All statistical analyses were performed using SAS (version 9.4), and a significance threshold set at a *p*-value < 0.05 was applied for all analyses. Descriptive analyses for categorical variables included summarizing the final dataset by frequency and proportion. For the continuous variable, long COVID duration, descriptive analyses included summarizing long COVID duration by mean and 95% confidence interval. Each covariate was stratified by long COVID experience, and the difference of each covariate variable between long COVID history groups was assessed using Pearson’s Chi-square tests or Fisher’s exact test (when more than 20% of cells had expected cell counts lower than 5).

Explanatory analyses were performed to identify factors associated with experiencing long COVID. First, univariate logistic regression models were used to assess the association between each selected covariate and experienced long COVID for the completed dataset. Categorical variables with insufficient numbers in one or more categories (e.g., education, COVID-19 vaccination history, COVID-19 vaccination type, province of residence) were not considered in explanatory analyses [45]. Additionally, based on our knowledge of the present study in the context of relevant literature, variables describing participant characteristics which were either minimally relevant to long COVID or captured in other covariates (e.g., place of birth, immigration status, length of stay in Canada) were not included in logistic regression. Similarly, categorical variables without reference levels facilitating meaningful comparisons (e.g., infection prevention efforts) were not considered in explanatory analyses. Overall, among covariate variables age group, gender, religiosity, marital status, work in health care, contact with the public at work, financial satisfaction, children or elderly in house, positive COVID-19 test results, COVID-19 symptom severity, COVID-19 treatment received, health status, underlying diseases, smoking status, regular alcohol consumption, COVID-19 vaccine side effects, and received Influenza vaccine were considered as predictive variables.

We used logistic LASSO regression to select the predictive variables most significantly associated with experiencing long COVID for inclusion in the final multivariate logistic regression model [46,47]. In logistic LASSO regression, the LASSO penalty function can increase prediction accuracy through reducing some coefficients to zero. Subsequently, multivariate logistic regression models were developed to yield odds ratios in the presence of the explanatory variables selected from the LASSO logistic regression model. Finally, sensitivity analysis consisted of creating multivariate logistic regression models for both the completed dataset and the imputed datasets to assess the potential influence of missing data on our parameter estimates. The Hosmer–Lemeshow test was then employed to evaluate the goodness of fit of our final multivariate logistic regression model [48]. Furthermore, sensitivity and specificity of the final model were assessed utilizing area under the ROC Curve, with a cut-point of ROC over 0.7 considered acceptable.

## 3. Results

### 3.1. Participants’ Sociodemographic and Health-Related Characteristics

Descriptive analyses characterize participants’ sociodemographic, COVID-related, and health-related characteristics stratified by long COVID. Each category is presented by frequency and column proportion, with a separate row for missing value frequency and overall proportion (see Table 1). Among 491 participants, 63 (12.83%) reported a long COVID experience, and 428 (87.17%) did not report any long COVID experience. Participants with a history of long COVID were more often women (69.84% in the long COVID group vs. 54.35% in the no long COVID group, *p* = 0.0207), religious (32.65% vs. 18.99%, *p* = 0.0275), with fair/poor/very poor health status (54.10% vs. 25.27%, *p* < 0.0001) and one or more underlying diseases (70.00% vs. 52.49%, *p* = 0.0116). Across long COVID groups, most participants were married/common law (84.13% vs. 80.14%, *p* = 0.4545) and residing in Ontario (90.48% vs. 82.44%, *p* = 0.1087). Further, more than half were 45 to 64 years old (63.49% vs. 59.81%, *p* = 0.8320), were not satisfied with their financial status (58.73% vs. 56.42%, *p* = 0.7313), had no children (aged ≤ 16) in their household (63.49% vs. 68.94%, *p* = 0.1302), and had no elderly (aged ≥ 65) in their household (68.25% vs. 69.97%, *p* = 0.9986). A small proportion of participants reported working in health care (6.35% vs. 12.22%, *p* = 0.1733) and contact with the public at work (25.81% vs. 25.76%, *p* = 0.9935).

### 3.2. Participants’ COVID-Related and Vaccine-Related Characteristics

As presented in Table 1, participants with long COVID experience more often had one (73.02% in the long COVID group vs. 44.10% in the no long COVID group) or two or more (14.29% vs. 1.79%) positive COVID-19 PCR or rapid antigen detection test results (*p* < 0.0001). Participants with long COVID experience also more often reported very serious/serious COVID-19 symptoms (46.03% vs. 22.50%, *p* = 0.0011) or COVID-19 vaccine side effects (51.67% vs. 30.91%, *p* = 0.0016) and had received TCM (23.81% vs. 8.67%) or prescription medicine (11.11% vs. 7.65%) for COVID-19 treatment (*p* = 0.0046), relative to those not reporting long COVID. Among both long COVID groups, most participants were nonsmokers (96.72% vs. 96.53%, *p* = 1.0000) and did not regularly consume alcohol (93.44% vs. 92.00%, *p* = 1.0000). Additionally, over half of the participants reported not receiving Influenza vaccination within the previous year (60.66% vs. 55.67%, *p* = 0.4664). Notably, although prevalence of three or more doses of COVID-19 vaccination was lower in the long COVID group (63.93%) compared to the no long COVID group (73.71%), this difference was insignificant (*p* = 0.1703).

### 3.3. Participants’ Long COVID Experience, Duration, Symptoms, and Underlying Diseases

Among the 63 participants (12.83%) reporting a history of long COVID, a high proportion (n = 51) also reported the duration of their corresponding COVID-19 symptoms. The mean duration of long COVID was 5.31 (95% CI: 4.06–6.57) months. A majority (n = 60) of participants reporting long COVID experience also identified the symptoms of COVID-19 they experienced for an extended period. The main symptoms of long COVID were difficulty concentrating (21.67%), pain/discomfort (15.00%), anxiety/depression (8.33%), fatigue (6.67%), shortness of breath (5.00%), and other symptoms (43.34%). Notably, other symptoms (n = 26) mainly included cough (30.77%), lethargy (7.69%), chest pain/tightness (7.69%), and hair loss (7.69%). Furthermore, most of the long COVID group (n = 42) reported at least one underlying disease, primarily including back/lumbar pain (40.48%), allergy (19.05%), diabetes (7.14%), cancer (7.14%), and obesity (7.14%).

### 3.4. Participant Characteristics Associated with Long COVID Experience

Several potentially significant associations with long COVID were identified from a univariate logistic regression model (see Table 2). Women had significantly higher odds of developing long COVID compared to men (OR = 1.945, 95% CI: 1.099–3.442, *p* = 0.0224). Similarly, individuals who identified as religious had greater odds of experiencing long COVID compared to non-religious individuals (OR = 2.068, 95% CI: 1.073–3.986, *p* = 0.0300). A history of multiple positive COVID-19 test results was strongly associated with long COVID, with odds increasing dramatically for individuals with two or more positive test results (OR = 33.907, 95% CI: 10.070–114.171, *p* < 0.0001). COVID-19 symptom severity also showed a significant association, as individuals reporting very serious or serious symptoms had increased odds of long COVID (OR = 6.809, 95% CI: 3.793–12.223, *p* < 0.0001). The odds of long COVID were also elevated among those who received prescription medicine (OR = 3.442, 95% CI: 1.345–8.808, *p* = 0.0099), over-the-counter medicine (OR = 2.746, 95% CI: 1.515–4.978, *p* = 0.0009), and TCM (OR = 7.555, 95% CI: 3.548–16.090, *p* < 0.0001) compared to those who received no treatment. Notably, good or very good health status appeared to be protective against long COVID experience (OR = 0.287, 95% CI: 0.165–0.500, *p* < 0.0001). Additionally, individuals with one or more underlying diseases had higher odds of long COVID (OR = 2.112, 95% CI: 1.172–3.808, *p* = 0.0129). Finally, experiencing COVID-19 vaccine side effects was positively associated with the odds of experiencing long COVID (OR = 2.389, 95% CI: 1.375–4.149, *p* = 0.0020).

Subsequently, we utilized multiple imputed datasets to estimate parameter coefficients for multivariate logistic regression analysis. Numerous significant associations with long COVID were identified from the imputed multivariate logistic regression model (see Table 3). Multivariate logistic regression models were utilized to produce odds ratios and 95% confidence intervals after a subset of explanatory variables were selected from the LASSO logistic regression model (see Supplemental Material—S2). Notably, in the imputed cases analysis, individuals with two or more positive COVID-19 test results had substantially higher odds of experiencing long COVID compared to those not reporting any positive test results (OR = 23.725, 95% CI: 5.098–110.398, *p* < 0.0001). Unsurprisingly, participants with one positive test result also exhibited increased odds of long COVID experience (OR = 4.286, 95% CI: 1.504–12.216, *p* = 0.0065). Although religiosity approached statistical significance in complete cases analysis, it fell short of the significance threshold for imputed cases analysis (OR = 2.257, 95% CI: 0.993–5.128, *p* = 0.0519). A significant positive association was observed between severe or very severe COVID-19 symptoms and the odds of long COVID experience (OR = 3.177, 95% CI: 1.160–8.702, *p* = 0.0246). Additionally, the use of TCM for COVID-19 treatment was strongly associated with higher odds of long COVID (OR = 8.259, 95% CI: 3.016–22.620, *p* < 0.0001), while the use of over-the-counter medications also showed a significant association (OR = 2.473, 95% CI: 1.035–5.909, *p* = 0.0416). Conversely, participants who reported very good or good health status had significantly lower odds of long COVID compared to those with fair, poor, or very poor health status (OR = 0.247, 95% CI: 0.112–0.544, *p* = 0.0005). However, no significant associations were observed for gender, working in health care, financial satisfaction, underlying diseases, or COVID-19 vaccine side effects in either analysis.

### 3.5. Sensitivity Analysis

A complete cases analysis was conducted using a dataset with no missing values (N = 333) for a multivariate logistic regression model (see Table 3). The results identified religiosity (OR = 2.611, 95% CI: 1.010–6.751, *p* = 0.0477), two or more positive COVID-19 test results (OR = 53.912, 95% CI: 6.901–421.189, *p* = 0.0001), one positive COVID-19 test result (OR = 7.328, 95% CI: 2.063–26.028, *p* = 0.0021), and the use of TCM for COVID-19 treatment (OR = 14.781, 95% CI: 4.006–54.542, *p* < 0.0001) as significant predictors of long COVID. Additionally, participants who reported very good/good health status were significantly less likely to develop long COVID (OR = 0.144, 95% CI: 0.055–0.378, *p* < 0.0001). While these findings largely align with the imputed data analysis, odds ratios for positive COVID-19 test results and TCM use were notably higher in the complete cases analysis compared to the multiple imputation approach, suggesting potential biases or variability in estimates arising from the exclusion of incomplete cases with missing observations.

Any discrepancies observed between complete cases analysis and imputed cases analysis could stem from reduced statistical power and potential biases associated with excluding participants with incomplete data [49]. For instance, the odds ratio for two or more positive COVID-19 test results was markedly higher in the complete cases analysis (OR = 53.912, 95% CI: 6.901–421.189) compared to the imputed cases analysis (OR = 23.725, 95% CI: 5.098–110.398). Similarly, TCM use had an elevated odds ratio in the complete cases analysis (OR = 14.781, 95% CI: 4.006–54.542) compared to the imputed cases analysis (OR = 8.259, 95% CI: 3.016–22.620). Under the assumption of data missing at random, imputed cases analysis can reduce such biases by incorporating information from incomplete cases into the analysis [49]. Therefore, despite the results of complete cases analysis offering valuable insights, the imputed cases analysis model likely provides more robust and reliable estimates for identifying predictors of long COVID from our data analysis.

## 4. Discussion

Nearly 13% of Canadian Chinese who participated in our study reported a history of long COVID, with an average duration slightly longer than 5 months. Among participants with a history of long COVID, most reported at least one underlying disease, with back/lumbar pain, allergy, diabetes, cancer, and obesity being among the most common (see Supplemental Material—S3). Additionally, the most frequently reported symptoms of long COVID were difficulty concentrating, pain/discomfort, anxiety/depression, fatigue, and shortness of breath (see Supplemental Material—S3). Our results also indicate that the number of positive COVID-19 test results, COVID-19 symptom severity, taking over-the-counter medicine, and receiving TCM were positively associated with long COVID among Canadian Chinese during the pandemic. Further, our results suggest a protective effect of very good/good health status.

### 4.1. Long COVID Experience, Duration, Symptoms, and Underlying Diseases

Findings from a meta-analysis of 120 studies present a wide range of long COVID prevalence estimates from the available literature, ranging from 0 to 93% [50]. Corresponding with this wide range was a pooled prevalence estimate of 42.1%. Such wide variation between studies may reflect commensurate variation in the definition and measurement of long COVID, perhaps diminishing comparability of results between different studies. Focusing on Canada, a retrospective chart review examining the experiences of a COVID-19 cohort in Toronto, Ontario showed that 27% of those infected with COVID-19 developed long COVID [51]. This is consistent with our findings, as about 26.9% of participants who reported at least one positive COVID-19 test result also reported experiencing long COVID. In another meta-analysis of 41 studies, the global prevalence of long COVID was again estimated to be as substantial as 43% [52]. Conversely, in a prospective observational cohort study of prolonged COVID-19 symptoms in the United Kingdom, 13.3% of 4182 incident cases of COVID-19 reported experiencing symptoms lasting at least 28 days [53]. This estimate is markedly lower than the long COVID prevalence among those with at least one positive test result (27%). Such disparities may be attributable to key differences in factors such as participant characteristics and regional differences in long COVID prevalence [31,50,52]. For example, the present study focuses on Canadian Chinese, while most available studies focus on general populations, likely differing in a variety of sociodemographic (e.g., age, gender), health-related (e.g., health status), and COVID-related (e.g., number of infections) factors [31,50,53]. These results suggest the prevalence of long COVID among Canadian Chinese was notable during the pandemic.

Another meta-analysis exploring the COVID-19 symptom durations reported in 15 papers described more than 50 COVID-19 symptoms persisting between 14 to 110 days post-infection [54]. In the Canadian context, a prospective population-based cohort study assessing acute COVID-19 symptoms and their evolution up to 9 months post-infection reported a long COVID duration of around 3 months [55]. This estimate is lower than the duration of long COVID of about 5 months reported in our study. Such a differentiation in long COVID duration may be attributable to differences in racial, behavioral, or lifestyle characteristics [56]. These differences could have been further influenced by disparate timing and the duration of data collection. Our study involved data collection between 22 December 2022, and 15 February 2023, whereas Benoit-Piau et al. (2023) recruited participants diagnosed with COVID-19 between 1 November 2020, and 31 May 2021, followed by a data collection period between August and September of 2021 [55]. Accordingly, future studies should further explore racial and ethnic differences in long COVID duration, in the Canadian context, to confirm whether these results represent a true increase in long COVID duration for Canadian Chinese relative to the general population.

Research conducted in the Chinese context has elucidated that long COVID can impact diverse organ systems, such as the respiratory system, nervous system, and digestive system, as well as mental health [20,29,30]. The most common symptoms of long COVID identified in the Chinese population were fatigue, cough, pharyngitis, lack of concentration, anxiety, myalgia, arthralgia, sputum, diarrhea, dyspnea, arrhythmias, fever, and hyperhidrosis. Similarly, the most common long COVID symptoms identified among the Chinese population in the Canadian context included difficulty concentrating, pain/discomfort, anxiety/depression, fatigue, and shortness of breath. These findings offer valuable insights into the impact of long COVID among Canadian Chinese, highlighting the diverse impairments and disruptions to daily functioning associated with the disease.

Concerning underlying diseases, previous studies underscored a higher prevalence of long COVID when the proportion of patients with diabetes, hypertension, obesity, respiratory diseases, liver disease, kidney disease, immune disorders, or allergies was greater [50]. Further, another review study stated heart disease, diabetes, cancer, COPD, chronic kidney disease, and obesity elevate the risk of both severe COVID-19 symptoms and long COVID [57]. The results of our study indicate allergy, diabetes, cancer, and obesity were common underlying diseases reported by the Canadian Chinese with a history of long COVID in our sample. In addition to these underlying diseases, the most reported comorbidity in our study was back/lumbar pain. This finding is also consistent with a growing body of literature suggesting that the incidence of back pain is increasing worldwide and may be accentuated during pandemic lockdowns [58,59,60].

### 4.2. Sociodemographic Characteristics, Health-Related Factors, and Long COVID

The present study failed to observe any significant association between long COVID experience and factors such as age group, marital status, work in health care, contact with the public at work, financial satisfaction, children or elderly in the household, smoking status, or regular alcohol consumption. This is inconsistent with a large population-based survey conducted by Wong et al. (2023) aiming to assess the COVID-19 experiences of 2712 COVID-19 patients across multiple centers in Beijing, Shanghai, Guangzhou, and Hong Kong during the pandemic [20]. Their findings highlighted the multifaceted nature of long COVID, revealing correlations between long COVID susceptibility, such as femininity, engagement in transportation or disciplined labor, living arrangements, smoking habits, overall health perception, presence of chronic diseases, medication use, and COVID-19 severity. Similarly, in their meta-analysis of 120 studies, Woodrow et al. (2023) identified an elevated prevalence of long COVID reported in studies wherein study samples had higher proportions of those older than 50 years of age, men, and people of non-White ethnicity [50]. Conversely, consistent with our findings, Woodrow et al. (2023) [50] did not identify an association between smoking status and long COVID in their systematic review. However, this was not directly addressed, and the power to detect that association may be too low, suggesting a need for future studies to further evaluate this association. Moreover, the systematic review of 50 studies from Chen et al. (2022) noted positive associations between long COVID and older age, number of COVID-19 symptoms, comorbidity, and pre-existing conditions such as obesity [52]. In addition, Subramanian et al. (2022) conducted a large retrospective matched cohort study using a United Kingdom-based primary care database to select 486,149 adults with a confirmed COVID-19 infection and no COVID-related hospitalization during 2022 [61]. They found the risk of long COVID was notably higher among older adults, females, ethnic minorities, those with lower socioeconomic status, smokers, and individuals with obesity or other comorbidities.

Although there was no significant association found between long COVID and factors including gender, religiosity, and underlying diseases in the final multivariate logistic regression model, these factors had significant effects on long COVID experience in our univariate models. Furthermore, religiosity approached significance in the complete cases analysis for multivariate regression. Employing a survey questionnaire developed by the United Kingdom’s Office of National Statistics, a 2022 population-representative survey of 3042 adults in the United States of America identified associations between long COVID and factors including female gender and underlying diseases [62]. Similarly, results from a study of 7150 COVID-19 patients in Spain found the probability of long COVID in women was significantly higher compared with men [15]. Intriguingly, a systematic review by David et al. (2023) outlines the potential relevance of religiosity as it pertains to individual responses to the COVID-19 pandemic, identifying numerous articles reporting that greater religiosity was associated with poorer adherence to public health behavior guidelines [63]. Relatedly, a multi-national comparison exploring regional variations in religiosity and the spread of COVID-19 during the pandemic revealed that declared attendance of religious services was associated with more infections and higher mortality [64]. Of particular importance, the observed association remained when controlling for regional variations in both the number of coronavirus tests per 1 million population and gross domestic product per capita.

The variation observed between the findings of the present study and those described above may be attributed to numerous factors [20,50,52,57]. First, differences in study design, sample size, and population characteristics could significantly influence observed associations. For example, while studies such as Wong et al. (2023) or Chen et al. (2022) utilized large and diverse samples covering multiple centers, our study may have been limited by a smaller or more homogenous sample, focusing on the GTA, likely reducing statistical power and the ability to detect certain associations [50,52]. Second, cultural and healthcare system differences may play a role, as factors such as healthcare access, diagnostic practices, and social determinants of health vary across regions and could influence the reported associations [31]. Third, differences in statistical modeling approaches, including adjustment for missing values, differences in analytical decisions (e.g., variable definition and categorization), the number of covariates, and the selected study sample [65]. Relatedly, it is difficult to isolate the respective contribution of any one predictor, such as gender, to the variance in long COVID. Each study differs in the inclusion and measurement of covariates when evaluating long COVID associated factors. This is exemplified by Shah et al. (2025) finding the association between female sex and long COVID decreased for their reduced Poisson regression model compared to their final model [66]. This indicates, when modelling long COVID, the inclusion of covariates downstream of gender may attenuate the association between long COVID and gender. Altogether, these considerations and the variations observed between available literature and the current study underscore the importance of contextualizing findings within methodological and regional frameworks of individual studies. We recommend that future research should continue to explore the complex interactions between individual demographic characteristics, health-related factors, and contextual factors influencing the likelihood of long COVID development.

### 4.3. Health Status and Long COVID

Among health-related factors assessed in our explanatory analyses, health status was significantly associated with long COVID. More specifically, the odds of long COVID were 75.3% lower for participants with very good or good health status compared to those with fair, poor, or very poor health status. A significant amount of the literature has reported similar results regarding a strong negative association between very good or good health status and the development of long COVID [20,67]. Consistent with our findings, using data from 10 longitudinal study samples in the United Kingdom, Thompson et al. (2022) completed a meta-analysis of survey responses from 6907 individuals with self-reported COVID-19 infection [68]. Notably, they found those with poor or fair pre-pandemic general health and mental health had a greater risk of long COVID. In a prospective, single-health-system, observational cohort study, Weerahandi et al. (2021) presented that patients (≥18 years of age) without good health conditions were more likely to experience long COVID [67]. Further, diverse studies have demonstrated that people with poor health status, such as those who had a history of hospitalization in an intensive care unit (ICU), had underlying diseases, or had multisystem inflammatory syndrome (MIS) during or after the COVID-19 infection, were at higher risk of developing long COVID [69,70,71].

### 4.4. Number of Positive COVID-19 Test Results and Long COVID

Remarkably, our results indicate a strong association between the number of positive COVID-19 test results and the likelihood of long COVID, such that participants with two or more positive test results were about 23.73 times more likely to develop long COVID compared to those not reporting positive test results. This is consistent with emerging literature describing a potentially important association between COVID-19 reinfection and long COVID. A 2023 narrative review article postulated the absolute number of long COVID outcomes substantially increased as a greater proportion of COVID-19 cases caused by the variety of Omicron family subvariants contributed to a considerable increase in COVID-19 reinfections [72]. Interestingly, a 2022 assessment of United States Department of Veterans Affairs databases explored the COVID-19 experience of a population ≥ 50 years of age, illustrating that the likelihood of developing long COVID significantly increased among individuals with reinfections compared to those with primary COVID-19 infections, regardless of COVID-19 vaccination status [73]. Moreover, Su et al. (2022) conducted a longitudinal multi-omic study on 309 COVID-19 patients, from diagnosis to convalescence, and found long COVID patients may also have lower antibody levels following infection, relative to those without long COVID experience, potentially elevating their risk of reinfection [74]. These results indicate effectively managing the burden of COVID-19 among vulnerable populations should incorporate strategies aimed at limiting reinfection [73].

### 4.5. COVID-19 Symptom Severity and Long COVID

As it pertains to COVID-19 symptom severity, participants in our study who experienced very serious or serious symptoms had approximately 3.18 times higher odds of developing long COVID compared to those who were asymptomatic or reported very mild symptoms. This result is consistent also with numerous previous studies that have identified symptom severity as a significant predictive risk factor for long COVID [15,20,57,67]. In April 2020, a prospective observational cohort study of 161 hospitalized patients ≥ 18 years of age with laboratory-confirmed COVID-19 diagnosis described how patients with more severe COVID-19 symptoms experienced poorer health status, which in turn increased the likelihood of long COVID [67]. Relatedly, Sudre et al. (2020) identified experiencing more than five symptoms in the first week of COVID-19 infection as being among the most important long COVID risk factors across gender and age groups [53]. Similarly, in their systematic review and meta-analysis, Woodrow et al. (2023) identified hospitalization and severity of COVID-19 infection as being the most important factors for long COVID experience [50]. In a multicenter cohort study examining 1969 inpatient and clinical records of individuals who had recovered from COVID-19 across five public hospitals in Spain, noting more than 60% of hospitalized COVID-19 survivors developed long COVID [15]. Collectively, these findings underscore the significant prevalence of long-lasting symptoms among those who have battled severe cases of COVID-19, highlighting the necessity for continued research, medical attention, and additional rehabilitation-oriented services, to address the lingering effects of COVID-19 beyond initial recovery.

### 4.6. COVID-19 Treatment Received and Long COVID

Additionally, our results underscore the significance of COVID-19 treatment received, with participants who reported treating symptoms with over-the-counter medicine having around 2.47 times higher odds of long COVID compared to those who did not treat symptoms. Remarkably, participants who treated symptoms with TCM were about 8.26 times more likely to develop long COVID compared to those not receiving treatment. To the best of our knowledge, the present study appears to be among the first to consider COVID-19 symptom treatment modalities as predictive risk factors for long COVID development. Around the time of data collection for our study, clinical guidelines primarily emphasized long COVID symptom management with various treatment approaches under active evaluation [9]. Research has highlighted the widespread use of self-prescribed medications for preventing and managing acute COVID-19, including antiretrovirals, antibiotics such as penicillin, vitamin C, traditional remedies, and drugs such as hydroxychloroquine [75,76,77]. The notable prevalence of self-prescribing behaviors observed during the pandemic were likely driven by the high morbidity and mortality associated with COVID-19, the scarcity of effective treatment guidelines, and limited access to health care during lockdowns. Importantly, self-prescription carries significant risks, including potential adverse drug interactions and the use of ineffective or even harmful therapies [78]. These findings suggest further research is warranted to fully understand the self-management practices that were used to manage COVID-19 symptoms; factors influencing their uptake; and their possible harms, particularly as it pertains to Canadian Chinese, who are less inclined to engage with Western medical services [27,28].

### 4.7. Vaccine-Related Factors and Long COVID

In contrast, our study failed to observe any significant association between history of long COVID and Influenza vaccination status. COVID-19 and Influenza, while being distinct types of infectious disease, have similarities in epidemiology, clinical manifestations, and pathological mechanisms [79]. Relatedly, some studies have shown that Influenza vaccination reduces the risk of COVID-19 infection and severity or mortality [80,81,82]. However, consistent with our results, existing studies indicate there is no significant effect of Influenza vaccination on subsequent long COVID experience.

A retrospective follow-up study of 1236 adults with long COVID found receiving a COVID-19 vaccination was significantly associated with prolonged COVID-19 symptoms for more than 1 year following initial infection [83]. Nonetheless, a recent systematic review describes numerous observational studies that have reported protective and therapeutic effects of COVID-19 vaccination on long COVID, including reductions in symptom severity, reinfections, and mortality [83,84]. Among our participants, we did not observe sufficient variation in COVID-19 vaccination status to conduct a robust analysis of such effects on the long COVID experience of Canadian Chinese. However, we explored the effect of vaccine side effects on long COVID. Despite COVID-19 vaccine side effects not reaching statistical significance in the final logistic regression model, vaccine side effects had a significant effect on long COVID history in univariate models. After reviewing other studies, it seems we are among the first to observe a potential association between long COVID and COVID-19 vaccine side effects [84]. Accordingly, we recommend future immunological studies assess vaccine side effects amongst long COVID patients.

### 4.8. Strengths and Limitations

This study has some important strengths. The study is among the first to offer insights into the long COVID experience of Chinese residents in Canada during the pandemic, indicating long COVID effected a notable proportion of Canadian Chinese, 12.83% in our sample. Furthermore, our study identifies key characteristics of Canadian Chinese who developed long COVID and factors associated with long COVID among Chinese residents in Canada. Such characteristics and factors include the number of positive COVID-19 test results, COVID-19 symptom severity, taking over-the-counter medicine, receiving TCM, and fair/poor/very poor health status. An important strength of our study pertains to the use of multiple imputed datasets to handle missing data and the use of LASSO multivariate logistic regression in model building. This approach to data analysis reduced bias and provided more robust and reliable parameter estimates for identifying factors significantly associated with long COVID experience in the present study [45,46,47,48,49]. Furthermore, the use of LASSO regression in model building accounted for potential multicollinearity among covariates such as health status, sociodemographic characteristics, number of positive COVID-19 test results, and COVID-19 symptom severity [30,57,73,85,86].

Despite these strengths, our study also has some notable limitations. Our use of a cross-sectional design precludes evaluation of time trends or making inferences regarding causality. Another key limitation is the potential for sampling bias, considering our utilization of a convenience sampling approach limiting generalizability. Most of our participants were Chinese residents of Ontario, concentrated in the GTA, who are unlikely representative of the total population of Chinese residents in Canada. While convenience sampling limits generalizability, leveraging the diverse community networks of partnered Canadian Chinese organizations (e.g., CNIW) yielded a large sample of a hard-to-reach and understudied population. Moreover, collecting data through an online survey might have introduced selection bias, as individuals who did not have or use electronic devices, as well as those without internet, were either excluded or under-represented in our sample. Nonetheless, an online survey was among the most suitable data collection methodologies during the COVID-19 pandemic, especially given the ongoing public health response in Canada during data collection [34]. Finally, our reliance on self-report measures for data collection introduces the potential for various information biases such as recall and social desirability bias. Notably, participants self-reported whether they experienced physical or psychological symptoms more than 12 weeks after initial infection, and these responses were used to derive long COVID experience. This outcome classification procedure may not have been consistent with our established definition of long COVID [10]. Inaccurate classification of long COVID experience may have skewed the results of the current study. Nonetheless, long COVID is a relatively recent condition, and its symptoms are often prominent and memorable. Moreover, there is no universally accepted clinical definition, and most existing studies rely on self-reports [87,88,89]. Some predictor variables (e.g., positive COVID-19 test results, COVID-19 symptom severity, and COVID-19 treatment received) may also be subject to recall bias. However, these misclassifications are likely to be non-differential, leading to conservative estimates of association.

## Figures and Tables

**Table 1:** Participants’ characteristics by long COVID experience.

		Long COVID		
Variables	N	Yes (n = 63)	No	*p*-Value
Age group				0.8320
65 or above	97	12 (19.05)	85 (19.86)	
45 to 64	296	40 (63.49)	256 (59.81)	
Under 45	98	11 (17.46)	87 (20.33)	
Missing	-	-	-	
Gender				0.0207
Women	275	44 (69.84)	231 (54.35)	
Men	213	19 (30.16)	194 (45.65)	
Missing	3 (0.61)	-	3	
Religiosity				0.0275
Religious	80	16 (32.65)	64 (18.99)	
Not religious	306	33 (67.35)	273 (81.01)	
Missing	105 (21.38)	14	91	
Marital status				0.4545
Married/common law	396	53 (84.13)	343 (80.14)	
Single/divorced/widowed	95	10 (15.87)	85 (19.86)	
Missing	-	-	-	
Work in health care				0.1733
Yes	53	4 (6.35)	49 (12.22)	
No	411	59 (93.65)	352 (87.78)	
Missing	27 (5.50)	-	27	
Contact with the public at work				0.9935
Yes	118	16 (25.81)	102 (25.76)	
No	340	46 (74.19)	294 (74.24)	
Missing	33 (6.72)	1	32	
Financial satisfaction				0.7313
Satisfied	199	26 (41.27)	173 (43.58)	
Not satisfied	261	37 (58.73)	224 (56.42)	
Missing	31 (6.31)	-	31	
Province of residence				0.1087
Ontario	409	57 (90.48)	352 (82.44)	
Others	81	6 (9.52)	75 (17.56)	
Missing	1 (0.20)	-	1	
Children (aged ≤ 16) in house				0.1302
Yes–two or more	53	12 (19.05)	41 (10.35)	
Yes–one	93	11 (17.46)	82 (20.71)	
No	313	40 (63.49)	273 (68.94)	
Missing	32 (6.52)	-	32	
Elderly (aged ≥ 65) in house				0.9986
Yes–two or more	81	11 (17.46)	70 (16.55)	
Yes–one	66	9 (14.29)	57 (13.48)	
No	339	43 (68.25)	296 (69.97)	
Missing	5 (1.02)	-	5	
Positive COVID-19 test results				<0.0001
Two or more	16	9 (14.29)	7 (1.79)	
One	218	46 (73.02)	172 (44.10)	
None/not sure	219	8 (12.70)	211 (54.10)	
Missing	38 (7.74)	-	38	
COVID-19 symptom severity				0.0011
Very serious/serious	74	29 (46.03)	45 (22.50)	
Mild	135	21 (33.33)	114 (57.00)	
Asymptomatic/very mild	54	13 (20.64)	41 (20.50)	
Missing	-	-	-	
COVID-19 treatment received				0.0046
Prescription medicine	22	7 (11.11)	15 (7.65)	
Over-the-counter medicine	82	20 (31.75)	62 (31.63)	
Traditional Chinese medicine	32	15 (23.81)	17 (8.67)	
No treatment	123	21 (33.33)	102 (52.04)	
Missing	4 (1.52)	-	4	
Health status				<0.0001
Very good/good	309	28 (45.90)	281 (74.73)	
Fair/poor/very poor	128	33 (54.10)	95 (25.27)	
Missing	54 (11.00)	2	52	
Underlying diseases				0.0116
One or more	232	42 (70.00)	190 (52.49)	
None	190	18 (30.00)	172 (47.51)	
Missing	69 (14.05)	3	66	
Smoking status				1.0000 ^a^
Smoker	15	2 (3.28)	13 (3.47)	
Nonsmoker	421	59 (96.72)	362 (96.53)	
Missing	55 (11.20)	2	53	
Regular alcohol consumption				1.0000 ^a^
Yes	34	4 (6.56)	30 (8.00)	
No	402	57 (93.44)	345 (92.00)	
Missing	55 (11.20)	2	53	
COVID-19 vaccination history				0.1703 ^a^
Three or more	325	39 (63.93)	286 (73.71)	
Vaccinated twice	109	20 (32.79)	89 (22.94)	
Vaccinated once	3	1 (1.64)	2 (0.52)	
Never vaccinated	12	1 (1.64)	11 (2.84)	
Missing	42 (8.55)	2	40	
COVID-19 vaccine side effects				0.0016
Yes	146	31 (51.67)	115 (30.91)	
No/not sure	286	29 (48.33)	257 (69.09)	
Missing	47 (9.81)	3	44	
Received Influenza vaccine				0.4664
Yes	192	24 (39.34)	168 (44.33)	
No	248	37 (60.66)	211 (55.67)	
Missing	51 (10.39)	2	49	

^1^ Chi-square tests (or Fisher’s exact test ^a^ when greater than 20% of the cells had expected cell counts less than 5) were utilized to evaluate and compare the distribution of categorical covariate variables between long COVID experience groups.

**Table 2:** Characteristics associated with long COVID experience from the univariate logistic regression model.

Variables	Long COVID		*p*-Value
	OR	95% CI	
Age group			
65 or above	0.949	0.485–1.860	0.8799
45 to 64	1.168	0.675–2.021	0.5777
Under 45	ref		
Gender			
Women	1.945	1.099–3.442	0.0224
Men	ref		
Religiosity			
Religious	2.068	1.073–3.986	0.0300
Not religious	ref		
Marital status			
Married/common law	1.313	0.642–2.688	0.4558
Single/divorced/widowed	ref		
Work in health care			
Yes	0.487	0.169–1.400	0.1817
No	ref		
Contact with the public at work			
Yes	1.003	0.544–1.849	0.9935
No	ref		
Financial satisfaction			
Satisfied	0.910	0.531–1.560	0.7314
Not satisfied	ref		
Children or elderly in house			
Yes	1.074	0.629–1.833	0.7937
No	ref		
Positive COVID-19 test results			
Two or more	33.907	10.070–114.171	<0.0001
One	7.054	3.242–15.346	<0.0001
None/not sure	ref		
COVID-19 symptom severity			
Very serious/serious	6.809	3.793–12.223	<0.0001
Mild	1.377	0.782–2.426	0.2675
Asymptomatic/very mild	ref		
COVID-19 treatment received			
Prescription medicine	3.442	1.345–8.808	0.0099
Over-the-counter medicine	2.746	1.515–4.978	0.0009
Traditional Chinese medicine	7.555	3.548–16.090	<0.0001
No treatment	ref		
Health status			
Very good/good	0.287	0.165–0.500	<0.0001
Fair/poor/very poor	ref		
Underlying diseases			
One or more	2.112	1.172–3.808	0.0129
None	ref		
Smoking status			
Smoker	0.944	0.208–4.290	0.9405
Nonsmoker	ref		
Regular alcohol consumption			
Yes	0.807	0.274–2.377	0.6974
No	ref		
COVID-19 vaccine side effects			
Yes	2.389	1.375–4.149	0.0020
No/not sure	ref		
Received Influenza vaccine			
Yes	0.815	0.469–1.415	0.4669
No	ref		

^2^ A significance threshold set at a *p*-value < 0.05 was applied. Abbreviations: OR = odds ratio, 95% CI = 95% confidence interval, ref = reference level or category used as baseline for each variable.

**Table 3:** Characteristics associated with long COVID experience from the final multivariate logistic regression model.

Variables	Complete Cases Analysis			Imputed Cases Analysis		
	OR	95% CI	*p*-Value	OR	95% CI	*p*-Value
Gender						
Women	1.431	0.590–3.470	0.4272	1.291	0.633–2.634	0.4820
Men	ref			ref		
Religiosity						
Religious	2.611	1.010–6.751	0.0477	2.257	0.993–5.128	0.0519
Not religious	ref			ref		
Work in health care						
Yes	0.300	0.049–1.818	0.1902	0.256	0.063–1.042	0.0570
No	ref			ref		
Financial satisfaction						
Satisfied	1.426	0.614–3.316	0.4091	1.500	0.747–3.013	0.2548
Not satisfied	ref			ref		
Positive COVID-19 test results						
Two or more	53.912	6.901–421.189	0.0001	23.725	5.098–110.398	<0.0001
One	7.328	2.063–26.028	0.0021	4.286	1.504–12.216	0.0065
None/not sure	ref			ref		
COVID-19 symptom severity						
Very serious/serious	2.739	0.840–8.924	0.0946	3.177	1.160–8.702	0.0246
Mild	0.344	0.097–1.222	0.0990	0.860	0.302–2.447	0.7758
Asymptomatic/very mild	ref			ref		
COVID-19 treatment received						
Prescription medicine	3.274	0.725–14.775	0.1229	2.969	0.868–10.156	0.0828
Over-the-counter medicine	1.956	0.682–5.608	0.2118	2.473	1.035–5.909	0.0416
Traditional Chinese medicine	14.781	4.006–54.542	<0.0001	8.259	3.016–22.620	<0.0001
No treatment	ref			ref		
Health status						
Very good/good	0.144	0.055–0.378	<0.0001	0.247	0.112–0.544	0.0005
Fair/poor/very poor	ref			ref		
Underlying diseases						
One or more	1.426	0.560–3.629	0.4570	1.609	0.751–3.445	0.2207
None	ref			ref		
COVID-19 vaccine side effects						
Yes	1.663	0.728–3.801	0.2275	1.738	0.823–3.668	0.1465
No/not sure	ref			ref		

^3^ A significance threshold set at a *p*-value < 0.05 was applied. Abbreviations: OR = odds ratio, 95% CI = 95% confidence interval, ref = reference level or category used as baseline for each variable.

## Data Availability

The datasets used and/or analyzed during this study are available from the corresponding author on reasonable request.

## Supplementary Materials

The following supporting information can be downloaded at https://www.mdpi.com/article/10.3390/biomedicines13040953/s1: Supplemental Material—S1: Characteristics of final dataset and study variable distributions; Supplemental Material—S2: LASSO variable selection models and sensitivity and specificity analyses; Supplemental Material—S3: Distribution of diseases reported by participants and symptoms reported by those with a history of long COVID.
