# Supplementary material for: Long COVID and Associated Factors Among Chinese Residents Aged 16 Years and Older in Canada: A Cross-Sectional Online Study
Source: Biomedicines. 2025 Apr 13;13(4):953. doi: 10.3390/biomedicines13040953 (PMC12024693; doi:10.3390/biomedicines13040953)
Supplement: Supplementary file 1 [file biomedicines-13-00953-s001.zip › biomedicines-3554311-supplementary.pdf]

Supplemental material – S1. Characteristics of final dataset and study variable distributions.

Table S1. Sample characteristics and study variable distributions.

**Variable**	**Frequency (n/N)**	**Percent (%)**
Age group		
65 or above	97/491	19.76%
45 to 64	296/491	60.29%
Under 45	98/491	19.96%
Missing	0/491	0.00%
Gender		
Women	275/491	56.01%
Men	213/491	43.38%
Missing	3/491	0.61%
Religiosity		
Religious	80/491	16.29%
Not religious	306/491	62.32%
Missing	105/491	21.39%
Marital status		
Married/common law	396/491	80.65%
Single/divorced/widowed	95/491	19.35%
Missing	0/491	0.00%
Education		
High school or below	13/491	2.65%
College/university	253/491	51.53%
Postgraduate	224/491	45.62%
Missing	1/491	0.20%
Place of birth		
Mainland China	473/491	96.33%
Others	18/491	3.67%
Missing	0/491	0.00%
Work in health care		
Yes	53/491	10.79%
No	411/491	83.71%
Missing	27/491	5.50%
Contact with the public at work		
Yes	118/491	24.03%
No	340/491	69.25%
Missing	33/491	6.72%
Financial satisfaction		
Satisfied	199/491	40.53%
Not satisfied	261/491	53.16%
Missing	31/491	6.31%
Immigration status		
Citizen/permanent resident	460/491	93.69%

Others	31/491	6.31%
Missing	0/491	0.00%
Length of stay in Canada		
Under 5 years	43/491	8.76%
5 to under 10 years	71/491	14.46%
10 years or above	377/491	76.78%
Missing	0/491	0.00%
Province of residence		
Ontario	409/491	83.30%
Others	81/491	16.50%
Missing	1/491	0.20%
Children (aged ≤ 16) in house		
Yes – two or more	53/491	10.79%
Yes – one	93/491	18.94%
No	313/491	63.75%
Missing	32/491	6.52%
Elderly (aged ≥ 65) in house		
Yes – two or more	81/491	16.50%
Yes – one	66/491	13.44%
No	312/491	63.54%
Missing	32/491	6.52%
Positive COVID-19 test results		
Two or more	16/491	3.26%
One	218/491	44.40%
None/not sure	219/491	44.60%
Missing	38/491	7.74%
COVID-19 symptom severity		
Very serious/serious	74/263	28.14%
Mild	135/263	51.33%
Asymptomatic/very mild	54/263	20.53%
Missing	0/263	0.00%
COVID-19 treatment received		
Prescription medicine	22/263	8.36%
Over-the-counter medicine	82/263	31.18%
Traditional Chinese medicine	32/263	12.17%
No treatment	123/263	46.77%
Missing	4/263	1.52%
Health status		
Very good/good	309/491	62.93%
Fair/poor/very poor	128/491	26.07%
Missing	54/491	11.00%
Underlying diseases		
One or more	232/491	47.25%
None	190/491	38.70%
Missing	69/491	14.05%

Infection prevention efforts		
Vitamin D	63/491	12.83%
Vitamin C	193/491	39.31%
Traditional Chinese medicine	30/491	6.11%
Others	65/491	13.24%
Missing	140/491	28.51%
Smoking status		
Smoker	15/491	3.06%
Nonsmoker	421/491	85.74%
Missing	55/491	11.20%
Regular alcohol consumption		
Yes	34/491	6.93%
No	402/491	81.87%
Missing	55/491	11.20%
COVID-19 vaccination history		
Three or more	325/491	66.19%
Vaccinated twice	109/491	22.20%
Vaccinated once	3/491	0.61%
Never vaccinated	12/491	2.45%
Missing	42/491	8.55%
COVID-19 vaccination type		
mRNA-type	381/479	79.54%
Vector	17/479	3.55%
Protein	1/479	0.21%
Inactivated virus	33/479	6.89%
Missing	47/479	9.81%
COVID-19 vaccine side effects		
Yes	146/479	30.48%
No/not sure	286/479	59.71%
Missing	47/479	9.81%
Received Influenza vaccine		
Yes	192/491	39.10%
No	248/491	50.51%
Missing	51/491	10.39%

Supplemental material – S2. LASSO variable selection models and sensitivity and specificity analyses.

We utilized the HPGENSELECT procedure in SAS (version 9.4) with selection method = LASSO (choose = AIC stop = AIC) to identify relevant variables for inclusion in the final logistic regression model. Based on selection frequency and coefficient strength across 10 imputed datasets, we included the following variables in the final model: (a) gender, (b) religiosity, (c) work in health care, (d) financial satisfaction, (e) positive COVID-19 test results, (f) COVID-19 symptom severity, (g) COVID-19 treatment received, (h) health status, (i) underlying diseases, and (j) COVID-19 vaccine side effects. **Table S2.1** displays both selected and unselected variables across 10 imputed datasets. Variables such as age group, marital status, contact with the public at work, smoking status, and regular alcohol consumption were not selected, suggesting low importance in predicting the outcome of interest. The model's goodness-of-fit was evaluated using the Hosmer & Lemeshow Goodness-of-Fit Test and the area under the Receiver Operating Characteristic (ROC) curve (**Table S2.2**). All p-values from the Hosmer & Lemeshow test were above 0.05 across unimputed and imputed datasets, indicating models were good-of-fitness. ROC values ranged from 0.8812 to 0.9150, demonstrating excellent to outstanding model discrimination.

Table S2.1. Logistic LASSO regression for model selection with imputed data.

**Fit statistic**	**Imputed dataset 1**	**Imputed dataset 2**	**Imputed dataset 3**	**Imputed dataset 4**	**Imputed dataset 5**	**Imputed dataset 6**	**Imputed dataset 7**	**Imputed dataset 8**	**Imputed dataset 9**	**Imputed dataset 10**
AIC	324.50	314.08	318.81	318.67	319.01	322.13	321.00	318.80	324.27	314.23
BIC	374.86	406.41	369.17	369.03	386.15	372.48	371.36	369.16	374.63	423.33
**Variable**										
Age group	-	-	-	-	-	-	-	-	-	-
Gender	-	0.0249	-	-	0.0001	-	-	-	-	0.0587
Religiosity	-	0.2736	-	-	-	-	-	-	0.0922	0.2088
Marital status	-	-	-	-	-	-	-	-	-	-
Work in health care	-	-0.0604	-	-	-	-	-	-	-	-0.1855
Contact with public at work	-	-	-	-	-	-	-	-	-	-
Financial satisfaction	-	-	-	-	-	-	-	-	-	0.0327
Children or elderly in house	-	-	-	-	-	-	-	-	-	-
Positive COVID-19 test results	0.3136	0.6734	0.3122	0.3167	0.4688	0.3185	0.3387	0.3113	0.2757	0.9565
COVID-19 symptom severity	0.5169	0.5794	0.5029	0.4962	0.5232	0.5096	0.4847	0.4979	0.5154	0.5733
COVID-19 treatment received	0.2941	0.5734	0.2792	0.2816	0.3587	0.2777	0.2722	0.2759	0.2982	0.6872

Health status	-0.3494	-0.4755	-0.4359	-0.3958	-0.4758	-0.3820	-0.3383	-0.4435	-0.3506	-0.5067
Underlying diseases	0.0966	-	-	-	-	-	-	-	-	0.2035
Smoking status	-	-	-	-	-	-	-	-	-	-
Regular alcohol consumption	-	-	-	-	-	-	-	-	-	-
COVID-19 vaccine side effects	0.0734	0.1501	0.0577	0.1311	0.0989	0.0576	0.1433	0.0435	-	0.2068
Received Influenza vaccine	-	-	-	-	-	-	-	-	-	-

Abbreviations: AIC = Akaike information criterion; BIC = Bayesian information criterion.

Table S2.2. Goodness-of-fit and ROC across unimputed and imputed models.

	**Complete Unimputed dataset**	**Imputed dataset 1**	**Imputed dataset 2**	**Imputed dataset 3**	**Imputed dataset 4**	**Imputed dataset 5**	**Imputed dataset 6**	**Imputed dataset 7**	**Imputed dataset 8**	**Imputed dataset 9**	**Imputed dataset 10**
Hosmer & Lemeshow Goodness-of-Fit Test[a]	0.9372	0.3883	0.2552	0.2710	0.5243	0.8614	0.7138	0.6829	0.4646	0.5911	0.9718
Area under ROC curve[b]	0.9150	0.8840	0.8962	0.8931	0.8907	0.8899	0.8874	0.8896	0.8942	0.8812	0.8897

[a] p-value > 0.05 indicates final model satisfies goodness-of-fit test.
[b] Area under the ROC curve represents ability of a fitted model to discriminate between participants with and without the outcome of interest. Area under the ROC curve between 0.8 and 0.9 indicates excellent discrimination, while area under the ROC curve over 0.9 indicates outstanding discrimination.
Abbreviations: ROC = Receiver Operating Characteristics.

Supplemental material – S3. Distribution of diseases reported by participants and symptoms reported by those with a history of long COVID.

Table S3.1. Distribution of participants' underlying diseases by long COVID experience.

Underlying disease	Total	Long COVID (n = 63)	No long COVID
Back/lumbar pain	59/491 (12.02%)	17/63 (26.99%)	42/428 (9.81%)
Allergy	32/491 (6.52%)	8/63 (12.70%)	24/428 (5.61%)
Hypertension	30/491 (6.12%)	1/63 (1.59%)	29/428 (6.78%)
Diabetes	23/491 (4.68%)	3/63 (4.76%)	20/428 (4.67%)
Obesity	16/491 (3.26%)	3/63 (4.76%)	13/428 (3.04%)
Osteoporosis	16/491 (3.26%)	2/63 (3.17%)	14/428 (3.27%)
Arthritis	15/491 (3.05%)	1/63 (1.59%)	14/428 (3.27%)
Cancer	11/491 (2.24%)	3/63 (4.76%)	8/428 (1.87%)
Heart disease	10/491 (2.04%)	2/63 (3.17%)	8/428 (1.87%)
Asthma	4/491 (0.81%)	0/63 (0.00%)	4/428 (0.93%)
Others	16/491 (3.26%)	2/63 (3.17%)	14/428 (3.27%)
None	190/491 (38.69%)	18/63 (28.58%)	172/428 (40.19%)
Missing	69/491 (14.05%)	3/63 (4.76%)	66/428 (15.42%)

Table S3.2. Distribution of symptoms reported by participants with a history of long COVID.

COVID-19 symptom	Frequency (n/N)	Percent (%)
Difficulty concentrating	13/63	20.63%
Pain/discomfort	9/63	14.29%
Anxiety/depression	5/63	7.94%
Fatigue	4/63	6.35%
Shortness of breath	3/63	4.76%
Memory problems	1/63	1.59%
Sleep disorder	1/63	1.59%
Others	24/63	38.10%
Missing	3/63	4.76%
